# Multiple regulatory events contribute to a widespread circular RNA downregulation in precancer and early stage of colorectal cancer development

**DOI:** 10.1186/s40364-025-00744-8

**Published:** 2025-02-20

**Authors:** Alessandro Camandona, Amedeo Gagliardi, Nicola Licheri, Sonia Tarallo, Giulia Francescato, Eva Budinska, Martina Carnogurska, Barbora Zwinsová, Barbara Martinoglio, Lorenzo Franchitti, Gaetano Gallo, Santina Cutrupi, Michele De Bortoli, Barbara Pardini, Alessio Naccarati, Giulio Ferrero

**Affiliations:** 1 Department of Clinical and Biological Sciences, University of Torino, Turin, 10100 Italy; 2 Italian Institute for Genomic Medicine (IIGM), c/o IRCCS Candiolo, Turin, 10060 Italy; 3 Candiolo Cancer Institute, FPO IRCCS, Candiolo, Turin, 10060 Italy; 4 RECETOX, Faculty of Science, Masaryk University, Brno, 61137 Czech Republic; 5 Institute of Biostatistics and Analyses, Faculty of Medicine, Masaryk University, Brno, 62500 Czech Republic; 6 Department of Surgery, “La Sapienza” University of Rome, Rome, 00161 Italy; 7 Department of Colorectal Surgery, Clinica S. Rita, Vercelli, 13100 Italy

**Keywords:** Circular RNAs, Colorectal cancer, Adenoma, Precancerous lesions, RNA-sequencing, RNA-binding proteins

## Abstract

**Background:**

Early detection of colorectal cancer (CRC) significantly improves its management and patients’ survival. Circular RNAs (circRNAs) are peculiar covalently closed transcripts involved in gene expression modulation whose dysregulation has been extensively reported in CRC cells. However, little is known about their alterations in the early phases of colorectal carcinogenesis.

**Methods:**

In this study, we performed an integrative analysis of circRNA profiles in RNA-sequencing (RNA-Seq) data of 96 colorectal cancers, 27 adenomas, and matched adjacent mucosa tissues. We also investigated the levels of cognate linear transcripts and those of regulating RNA-binding proteins (RBPs). Levels of circRNA-interacting microRNAs (miRNAs) were explored by integrating data of small RNA-Seq performed on the same samples.

**Results:**

Our results revealed a significant dysregulation of 34 circRNAs (paired adj. p < 0.05), almost exclusively downregulated in tumor tissues and, prevalently, in early disease stages. This downregulation was associated with decreased expression of circRNA host genes and those encoding for RBPs involved in circRNA biogenesis, including *NOVA1*, *RBMS3*, and *MBNL1*. Guilt-by-association analysis showed that dysregulated circRNAs correlated with increased predicted activity of cell proliferation, DNA repair, and c-Myc signaling pathways. Functional analysis showed interactions among dysregulated circRNAs, RBPs, and miRNAs, which were supported by significant correlations among their expression levels. Findings were validated in independent cohorts and public datasets, and the downregulation of circLPAR1(2,3) and circLINC00632(5) was validated by ddPCR.

**Conclusions:**

These results support that multiple altered regulatory mechanisms may contribute to the reduction of circRNA levels that characterize early colorectal carcinogenesis.

**Supplementary Information:**

The online version contains supplementary material available at 10.1186/s40364-025-00744-8.

## Background

Colorectal cancer (CRC) accounts for approximately 10% of all annually diagnosed cancers and it is the second leading cause of cancer-related deaths worldwide. The high lethality of CRC is often due to a late diagnosis, as the disease can progress asymptomatically until reaching advanced stages [1]. Typically, CRC develops from precancerous lesions, which are primarily classified as adenomas or serrated adenomas, and it can take several decades for these lesions to manifest into a detectable cancer [2]. Recent advancements in high-throughput technologies have enhanced the understanding of this disease. RNA sequencing (RNA-Seq) has allowed a better understanding of the cancer transcriptome helping to better describe the CRC molecular heterogeneity [3, 4]. Among the large spectrum of RNAs with altered regulatory activity in cancer cells, circular RNAs (circRNAs) have emerged as a class with distinctive molecular features [5, 6]. circRNAs are mono- or multi-exonic RNAs produced by a peculiar alternative splicing event, defined as back-splicing. In this process, the splice donor site is joined with an upstream splice acceptor site, resulting in a characteristic exon-exon junction, called back-splicing junction (BSJ) [7]. Due to their covalently closed structure, circRNAs are highly resistant to exonuclease digestion, making them stable in extracellular environments and detectable in different human biofluids [8]. Growing evidence suggests that circRNAs play an essential role in gene regulation by interacting with microRNAs (miRNAs) and some regulatory RNA-Binding Proteins (RBPs). They can act as miRNA sequesters (“sponge”), protein decoys, scaffolds, and recruiters, thus modulating gene function [7, 9]. Furthermore, circRNAs can be translated into short peptides [10, 11].

The circRNA dysregulation in tumors and the possibility of profiling this RNA class using total RNA-Seq data [12, 13] have sparked interest in exploring their expression pattern to identify novel disease biomarkers [14, 15]. Previous studies have reported that several circRNAs are dysregulated in CRC [16], with many researchers observing a decrease of these molecules that correlates with specific tumor characteristics [17, 18]. This widespread downregulation may be attributed to an impaired host gene expression or to the disruption of circRNA biogenesis, potentially due to altered expression of those RBPs involved in the circularization [19]. Lower circRNA levels have been observed in colorectal tumors with respect to both stroma and normal tissue, with their abundance reported to be higher in senescent cells than proliferating cells [20]. This decrease in proliferating cells may be partially explained by cytoplasmic dilution [20]. CircRNAs are stable molecules that persist in the cytoplasm for several hours, and cells generally produce tightly controlled copies of them [21]. During cell division, the cytoplasmic contents, including circRNAs, are divided between the daughter cells resulting in a reduced concentration without any actual transcriptional downregulation [20, 22]. The high rate of cell division, characteristic of tumorigenesis, could therefore contribute to the widespread circRNA downregulation observed in tumors. However, it is still unclear whether other molecular events associated with the early stages of tumor development also play a role in this observed circRNA decrease.

This study provides a comprehensive analysis of circRNA expression profiles from RNA-Seq data of tumor and adenoma tissues compared to matched adjacent mucosa, in a cohort of 123 patients with CRC or precancerous lesions. The analysis showed a widespread decrease in circRNA levels in both CRC and advanced adenoma samples. This reduction is accompanied by a downregulation of circRNA host genes and RBPs involved in circRNA biogenesis, suggesting that multiple factors contribute to circRNA decrease, beyond the effects of a higher cancer-related cell division alone. Furthermore, small RNA-Seq profiles from the same samples revealed specific alterations in circRNA-interacting miRNAs regulating signaling pathways associated with cell DNA-damage response and apoptosis.

## Materials and methods

### Study cohorts

The study comprises 123 Italian subjects recruited from the “Clinica Santa Rita” in Vercelli. Based on the colonoscopy results, participants were classified into patients with CRC (*n* = 96), advanced adenomas (AA, *n* = 24), and non-advanced adenomas (nAA, *n* = 3) (Table 1). No serrated adenomas were included in the cohort. Details of this cohort were previously described [23, 24]. Moreover, total RNA-Seq data of 159 CRC samples from the COLOBIOME cohort described in [25] were analyzed to evaluate the levels of the differentially expressed circRNAs detected in the Italian cohort.

**Table 1:** Study population characteristics

	Type of lesion		
	Tumor (*N* = 96)	Adenoma (*N* = 27)	*P*-value
**Age** Mean (SD)	69.1 (± 10.1)	69.6 (± 11.6)	0.73
**Sex (%)**			0.83
Male	56 (58.3%)	15 (55.6%)	
Female	40 (41.7%)	12 (44.4%)	
**BMI**			0.06
Mean (SD)	26.5 (± 5.3)	24.4 (± 3.8)	
Missing	10 (10.4%)	3 (11.1%)	
**Smoking habit (%)**			0.71
Never	44 (45.8%)	11 (40.7%)	
Former	12 (12.5%)	5 (18.6%)	
Smoker	31 (32.3%)	9 (33.3%)	
Missing	9 (9.4%)	2 (7.4%)	
**Stage (%)**			
nAA	-	3 (11.1%)	
AA	-	24 (88.9%)	
0-I	26 (27.1%)	-	
II	32 (33.3%)	-	
III	31 (32.3%)	-	
IV	3 (3.1%)	-	
Missing	4 (4.2%)	-	
**Mucinous status (%)**			
nmCRC	70 (56.9%)	-	
mcCRC	20 (16.2%)	-	
mCRC	6 (4.9%)	-	
Missing	27 (22%)	-	
**CMS (%)**			
CMS1	8 (8.3%)	-	
CMS2	27 (28.1%)	-	
CMS3	24 (25.0%)	-	
CMS4	23 (24.0%)	-	
Missing	14 (14.6%)	-	
***APC* mutational status (%)**			0.24
Mutated	55 (57.3%)	18 (66.7%)	
Wild-Type	38 (39.6%)	6 (22.2%)	
Missing	3 (3.1%)	3 (11.1%)	
***KRAS* mutational status (%)**			1.00
Mutated	25 (26.0%)	7 (25.9%)	
Wild-Type	68 (70.8%)	18 (66.7%)	
Missing	3 (3.2%)	2 (7.4%)	

*BMI* Body Mass Index, *nAA* non-advanced adenoma, *AA* advanced adenoma, *CRC* colorectal cancer, *CMS* Consensus Molecular Subtype, *nmCRC* non mucinous CRC, *mcCRC* CRC with mucinous component (5%−50%), *mCRC* mucinous CRC (> 50%), *SD* standard deviation

The local ethics committees of Azienda Ospedaliera SS. Antonio e Biagio e C. Arrigo of Alessandria (Italy, protocol no. Colorectal miRNA CEC2014), Masaryk Memorial Cancer Institute (protocol no. 2018/865/MOU), and Masaryk University of Brno (Czech Republic, protocol no. EKV2019-044) approved the study. All patients gave written informed consent following the Declaration of Helsinki before participating in the study.

### Sample collection and nucleic acids extraction

For all subjects, paired tumor/adenoma tissue and adjacent non-malignant mucosa (at least 20 cm distant) were obtained from CRC and adenoma patients during surgical resection and immediately immersed in RNAlater solution (Ambion). All samples were stored at −80 °C until use. From each tissue sample, a piece of 5–10 mg was diced and homogenized using an ULTRA-TURRAX® homogenizer (Janke and Kunkel IKA). Homogenate tissues were processed for RNA extraction using QIAzol® (QIAGEN) and following the manufacturer’s instructions.

To remove all traces of DNA, purified RNA samples were then cleaned up and DNase-treated with the RNA Clean & Concentrator™−5 kit (Zymo Research) following the manufacturer`s protocol. The obtained total RNA was quantified with Qubit™ 4 fluorometer using Qubit™ RNA Broad range Assay Kit (Invitrogen), and the quality of the input RNA was determined by RNA Integrity Number (RIN) measurement obtained by running the samples on an Agilent Bioanalyzer® RNA 6000 Nano chip (Agilent Technologies).

DNA was extracted from tumor/adenoma and adjacent mucosa tissues for mutational profiling and processed as described in Gagliardi et al., 2023 [23]. After a homogenization step (Promega homogenization solution), the tissue samples were processed using the Maxwell RSC Tissue DNA Kit (Promega). Before loading samples onto Maxwell RSC Cartridges, 300 µL of Lysis Buffer and 30 µL of Proteinase K were added to the homogenized samples and further incubated for 20 min at 56 °C. DNA was quantified with Qubit dsDNA High Sensitivity Assay Kit (Invitrogen).

### Library preparation for total and small RNA sequencing

Details on the RNA-Seq protocols and analysis were previously described [23, 24]. Briefly, for total RNA-Seq, a starting quantity of 500 ng of total RNA was subjected to ribosomal RNA (rRNA) removal using the NEBNext® rRNA Depletion Kit v2 (New England Biolabs) following manufacturer protocol. 1–100 ng of rRNA-depleted RNA was then used as input for library preparation using the NEBNext® Ultra II Directional RNA Library Prep Kit for Illumina® (New England Biolabs) according to the manufacturer’s instructions and with the necessary modifications according to the RIN assessment of starting material.

The RNA fragments previously generated through heat treatment and hybridized with random primers were reverse transcribed to first strand cDNA. Following the first strand, the second strand of cDNA was synthesized by incorporating dUTP. To ensure an efficient adapter ligation, the ends of the cDNA molecules were prepared through terminal repair and single adenylation at the 3’ end. Following the excision of the uracil-containing strand, adapter-ligated fragments of each library were PCR-enriched with unique indexed primers. After PCR amplification, cDNA libraries were pooled and purified; the yield and quality were assessed using Qubit™ dsDNA HS Assay Kit (Invitrogen) and Agilent® High Sensitivity DNA Kit (Agilent Technologies), respectively. Libraries were captured on Illumina® flow cells and paired-end sequenced (100 bp-long reads) on Illumina® NovaSeq™ 6000 Sequencer (Illumina), according to the manufacturer’s instructions.

For small RNA sequencing, the details on protocols and analysis were elsewhere described [23, 24].

### Library preparation for mutational profiling (TruSight Oncology 500)

DNA libraries were prepared using the hybrid capture-based TruSight Oncology 500 High-Throughput (TSO500-HT) Library Preparation Kit (Illumina). In brief, the genomic DNA was fragmented using the Covaris ME220 focused-ultrasonicator (Covaris) for 10 s at 50 W. After end repair, A-tailing, and adapter ligation, the adapter-ligated fragments were amplified using primers to add index sequences for sample multiplexing. Libraries were enriched through two hybridization/capture steps using specific probes: a pool of oligos specific to 523 genes targeted by TSO500-HT was hybridized to the targeted regions. The final steps were PCR amplification, clean-up, and quantification with Qubit dsDNA HS Assay Kit (Invitrogen). Following pooling and denaturation, libraries were diluted to the appropriate loading concentration and finally sequenced on Illumina NovaSeq 6000 Sequencer (Illumina).

### RNA-Seq data analysis and circRNA dataset definition

FastQ files from RNA-Seq were pre-processed and quality-controlled using FastP v.0.21 in default conditions [26]. Then, reads mapping on rRNA fragments were excluded using SortMeRNA v.4.3.6 [27]. Surviving reads were aligned on GENCODE 40 annotation using STAR v2.5.1b [28] in default settings. Transcript levels were quantified using RSEM v1.3.0 [29].

CIRI2 v2.1.1 [30] was applied to quantify circRNA levels. Precisely, reads were aligned first on the human genome using bwa v0.7.17 [31]; then, the read alignment files were used as input of CIRI v2.06 that was applied with default setting for circRNA prediction. Only circRNAs associated with at least two BSJ supporting reads were considered for the analysis. In all circRNA analyses presented here, the circRNA levels were expressed as the number of BSJ reads.

To filter circRNAs characterized by low expression levels and detected only in very few samples, the *NearZeroVar* function of the R *caret* package v6.0–94 was applied. This function computes the mode of its levels across the analyzed subjects for each analyzed feature. Then, it removes all the features characterized by the lowest variance with respect to this value. In our dataset, the most frequent value for most circRNAs was 0 (i.e., no circRNA expression). Therefore, all the circRNAs detected in a few samples and characterized by a minimum BSJ supporting reads were removed. The filtered circRNA read count table was normalized using the R package *DESeq2* v1.38.3 [32]. CircRNA annotation has been decided according to the method proposed in Chen et al., 2023 [33]. Specifically, the prefix “circ” followed by the gene symbol and the exon order within brackets were used.

The miRNA profiling in tumor tissue was performed as previously described [24]. Briefly, small RNA-Seq reads were aligned on human hairpin miRNA sequences from miRbase v22.1 using the Docker4Seq analysis pipeline [34]. Normalization of miRNA levels and differential analysis were performed using DESeq2.

### Differential expression analysis

Paired differential expression analysis for circRNAs and other transcripts was computed using *DESeq2* v1.38.3 library, matching lesion samples with their adjacent mucosa samples. Those circRNAs characterized by a median BSJ reads > 10 in at least one sample class and with an adjusted *p*-value (adj. p) < 0.05 were considered as differentially expressed.

RBP-coding genes were selected considering the EMBL RBPBase gene set (v0.2.1, https://apps.embl.de/rbpbase/). Only the genes whose product is a validated RBP (protein-coding gene and known RBP-coding gene, *n* = 1,513) were selected. Tximport R package was applied to normalize the expression levels of these genes (transcripts per million, TPM), while DESeq2 was used for the paired differential expression analysis. Those genes with median TPM > 1 in at least one sample group and with an adj. p < 0.05 were considered differentially expressed.

### Droplet digital polymerase chain reaction (ddPCR™)

Following the manufacturer's instructions, RNA from CRC tissues (500 ng) was retrotranscribed in cDNA using the Reliance Select cDNA Synthesis Kit (Bio-Rad).

The resulting cDNA was 10 × diluted and used for running ddPCR experiments. The PCR mix was prepared with 11 μL of 2X QX200 ddPCR EvaGreen supermix, 7.9 μL of nuclease-free water, 0.55 μL of each circRNA-specific primer (forward (FW) and reverse (RV) (see Table 2 for primer sequences). The final concentration of both primers was 200 nM. To the 20 μL of the working mix, 2 μL of cDNA was added to the reaction in a 96-well plate.

**Table 2:** CircRNA back-splicing sequences and ddPCR primers

circRNA ID	Position	Synthesis sequence	primer FW	primer RV
circVRK1(2,11)	chr14:96,833,467–96,860,735	AAATTTACGTGACATTCTTTTGCAAGGACTAAAAGCTATAGGAAGTAAGGATGATGGCAAATTGGACCTCAGTGTTGTGGAGAATGGAGGTTTGAAAGCAA**AAACAATAACAAAG**TGAAAATGCCTCGTGTAAAAGCAGCTCAAGCTGGAAGACAGAGCTCTGCAAAGAGACATCTTGCAGAACAATTTGCAGTTGGAGA	AAGGATGATGGCAAATTGGA	CTCTTTGCAGAGCTCTGTCT
circLPAR1(2,3)	chr9:110,972,073–110973558	AAAATTTGTCTCCCGTAGTTCTGGGGCGTGTTCACCACCTACAACCACAGAGCTGTCATGGCTGCCATCTCTACTTCCATCCCTG**TAATTTCACAGCCCCAG**GTGGACGTCTGATTTATGAAGCTCCCCATCCACCTATCTGAGTACCTGACTTCTCAGGACTGACACCTACAGCATCAGGTACACAGCTTCTCCTAGCA	GTAGTTCTGGGGCGTGTTCA	AAGTCAGGTACTCAGATAGG
circLINC00632(5)	chrX:140,783,175–140784659	GTCTTCCATCAACTGGCTCAATATCCATGTCTTCCAACGTCTCCAGTGTGCTGATCTTCTGACATTCAGGT**CTTCCAGTGTCTGCAATATCCAG**GGTTTCCGATGGCACCTGTGTCAAGGTCTTCCAACAACTCCGGGTCTTCCAGCGACTTCAAGTCTTCCAATAATCTCAA	TCTTCCATCAACTGGCTCAA	ATTGGAAGACTTGAAGTCGC

Sequences generated for each analyzed circRNA and the forward and reverse ddPCR primers used for the analysis. The exonic sequences downstream and upstream of the BJS are reported in bold and underlined, respectively

Droplets were generated with the QX200 AutoDG Droplet Digital PCR System for EvaGreen-based Droplet Digital PCR (ddPCR). For each droplet generator run, ddPCR reactions for samples were prepared in duplicate or in single and for each run we included negative template controls and RT controls. The amplification conditions were run on a T100 thermal cycler (Bio-Rad) with a temperature ramp rate set to 2.5 °C/s, with the lid heated to 105 °C, and according to the Bio-Rad recommendations (5 min at 95 °C, 40 cycles of a two-step thermal profile of 30 s at 95 °C for denaturation, and 60 s at 60 °C, followed by 5 min at 4 °C and a final hold at 90 °C for 5 min for droplet stabilization, and cooling to 4 °C).

After PCR, the plates were transferred to a QX100 droplet reader (Bio-Rad). Data acquisition was performed with QuantaSoft™ (1.6.6.0320; Bio-Rad). The rejection criterion for the exclusion of a reaction from subsequent analysis was a low number of droplets measured (< 10,000 per 20 μL PCR). The data from the ddPCR are given in target copies/μL reaction.

### Analysis of public datasets

Two public datasets of circRNA expression in primary CRC and adjacent mucosa, measured by cDNA capture RNA-Seq [17] and NanoString (GSE233799) [20], were also included in the study and analyzed. Data from the first study were retrieved from the supplementary material of the manuscript, while the second dataset was analyzed with GEO2R. CircRNA quantification data from 30 tumor paired with adjacent mucosa tissue samples were retrieved from supplementary material of Li et al. study [15] and included in the analysis.

The prediction of single-cell expression of the circRNAs was retrieved from the TMECircDB [17] data set. The circRNA host gene and RBP levels analysis was performed considering public RNA-Seq datasets retrieved from the NCBI GEO portal (https://www.ncbi.nlm.nih.gov/geo/). Specifically, nine different datasets were selected, including both colorectal tumor (GSE156451, GSE142279, GSE95132, GSE74602, GSE41657, GSE20916) and adenoma (GSE20916, GSE8671, GSE77953, GSE4183) samples matched with adjacent normal tissue. Single cell expression analysis was performed considering the processed data provided in GSE132465 and considering the TPM-normalized expression levels [35]. A summary of the analyzed datasets is reported in Table 3.

**Table 3:** Analyzed cohorts and public datasets

Data	Type of data	Source	Adjacent tissue samples (n)	Adenoma tissue samples (n)	CRC tissue samples (n)
Cohorts	RNA-Seq	COLOBIOME cohort (Zwinsová et al., 2021)	-	-	159
		This study	123	27	96
Public datasets		GSE156451	80	-	80
		GSE142279	20	-	20
		GSE95132	10	-	10
		GSE74602	30	-	30
		GSE41657	12	-	25
		GSE20916	44	-	46
			44	45	-
		GSE8671	32	32	-
		GSE77953	16	20	-
		GSE4183	8	15	-
		TMECircDB/GSE221240	8	-	6
		Li et al., 2024 (Supplementary material)	30	-	30
	NanoString (microarray-based)	GSE233799	10^a^	-	10^b^
	Single-cell RNA-Seq	GSE132465	10	-	25

^a^ = Normal epithelium (*n* = 5); Normal stroma (*n* = 5); ^b^ = Tumor stroma (*n* = 5); Tumor (*n* = 5)

### CircRNA functional analysis

The functional analysis of circRNAs was performed following a guilt-by-association approach relating the circRNA levels with single-sample score of specific pathways. Specifically, the analysis of MSigDB hallmark gene sets [36] was computed using the *singscore* v1.18.0 [37] and *GSEAbase* v1.60.0 libraries. Moreover, to inspect the difference of pathway scores in lesions with respect to adjacent mucosa samples, a log2FC of the computed scores was calculated. All the associations with an adj. *p* < 0.001 and a coherent correlation coefficient (|ρ|> 0.30) among sample classes were analyzed.

The list of miRNAs and RBPs interacting with the differentially expressed circRNAs was downloaded from *CircNET* v2.0 [38] and *riboCirc* [39]. The interactions with miRNAs were filtered according to the number of databases in which the interaction was detected (at least two over three). The RBP interactions were selected according to the predicted interactions only occurring in circularizing exons. A Spearman correlation analysis was computed among levels of interacting circRNAs, miRNAs, and RBPs. The interactions supported by a significant correlation (adj. *p* < 0.05) were considered for further analysis.

miRNA and validated miRNA-target gene sets were downloaded from miRTarBase (https://mirtarbase.cuhk.edu.cn, update 2022). Enrichment scores were computed using the *RBiomirGS* package v0.2.19 as previously described [24].

### Statistical and computational methods

Statistical analyses were computed in R v4.2.2 with the integration of *DESeq2* v1.38.3 and *ggpubr* v0.6.0 libraries. Correlation analyses were performed using the *Hmisc* v5.1–1 library and the Spearman method. The associations with Benjamini-Hochberg (BH)-adjusted *p* < 0.05 were considered significant. Correlation tests for functional characterization were initially computed for all the samples (total) and then stratified for a sample class. The associations with adj. *p* < 0.05, coherent ρ among the sample classes, and supported by predicted interaction were considered.

The analysis of candidate RBP regulating circRNA biogenesis was performed considering the RBP set from the EMBL dataset [40]. RBP interactions were predicted and visualized using STRING v.12.0 (https://string-db.org). The analysis of the RBP motif in the proximity of circularizing exons was performed by scanning a region of ± 50 bp centered on the splice sites involved in the BS events. The motif finding analysis was performed using RBPmap [41] with options "high stringency" (*P*-value[significant] < 0.005 and *P*-value[suboptimal] < 0.01) and conservation filter equal to true. Other statistical tests for continuous variables were performed using the Wilcoxon rank sum test.

The survival analysis was performed using the *survminer* v0.5 R package, by stratifying CRC patients based on the median levels of each circRNA.

## Results

### Demographic and clinical information of the study population

CircRNA levels were derived from RNA-Seq data of tumor and adenoma tissues matched with adjacent colonic mucosa from 123 Italian patients (IT-cohort, Table 1). Specifically, this study population comprised 96 CRC patients, including 56 males (58%) and 40 females (42%), with an average age of 69.1 years old (y.o.) (range = 48–88 y.o.). According to the American Joint Committee on Cancer (AJCC), tumors from CRC patients were classified in stages 0-I (*n* = 26), stage II (*n* = 32), stage III (*n* = 31), and stage IV (*n* = 3). For four patients, the stage could not be assessed. Adenoma patients comprised 15 males (52%) and 12 females (48%), with an average age of 70 y.o. (range = 42–93 y.o.). Twenty-four subjects had an advanced adenoma (AA), while three had a non-advanced adenoma (nAA). The cohort included 40 smokers, 17 former smokers, and 55 non-smokers. Two patients were underweight (BMI < 18.5), 35 were overweight (25 < BMI < 30), 19 were obese (BMI ≥ 30), and 57 had normal weight (Table 1). CRC and adenoma patients were comparable in terms of age, sex, BMI, smoking status, *APC* and *KRAS* mutational status.

### CircRNA levels are reduced in tumor tissue compared to adjacent mucosa

**Fig. 1:**
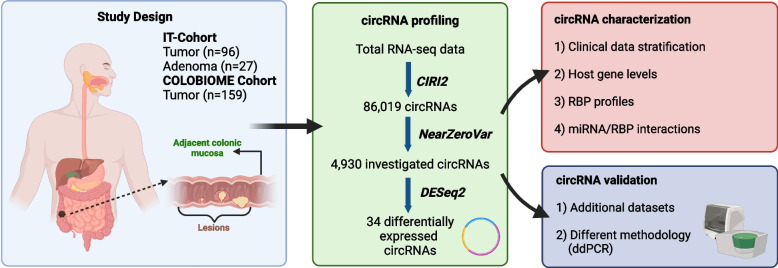
Summary of the study design reporting the sample size and workflow of the analyses

The analysis of the RNA-Seq data from 123 tissue pairs from the IT-cohort resulted in the identification of a total of 86,019 circRNAs, most of which (*n* = 52,031, 60.5%) exhibited low abundance and were detected only in one sample (Fig. 1 and Figure S1A). Focusing only on filtered molecules (see Material and Methods for details), 4,930 circRNAs were further investigated (Table S1A). This set included 4,645 exonic (94%), 196 intronic (4%), and 89 intergenic (2%) circRNAs (Table S1A). Among them, an average of 1,345 (range 154–3,067) circRNAs were detected in each sample with chr14:105,587,554|105,707,562, circLINC00632(5), and chr14:105624896|105643525 globally reporting the highest expression levels (Table S1A). Globally, the number of circRNAs was significantly lower in tumors (mean = 1,217) with respect to adjacent mucosa (mean = 1,456) (Figure S1B).

**Fig. 2:**
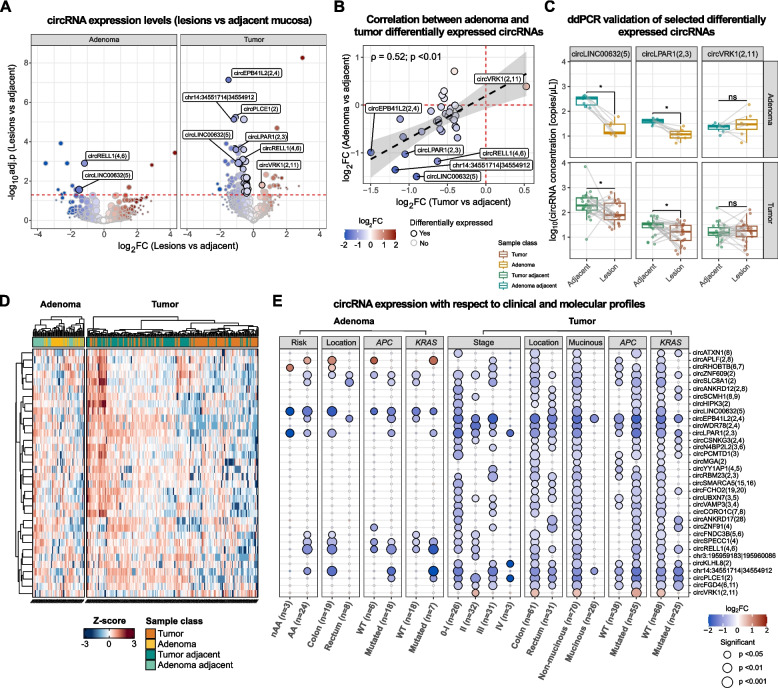
CircRNA levels are depleted in lesion samples with respect to adjacent mucosa. **A** Volcano plots of the circRNA paired differential analysis reporting on the y-axis the -log10 adj. p-value and on the x-axis the extent of expression change (as log2FC) between lesion (adenoma on the left, and tumor on the right) and adjacent mucosa samples. Dot size is proportional to circRNA median expression. The red dashed line indicates the adj. p-value threshold of 0.05. Black dot border highlights the differentially expressed circRNAs. The labels identify the most significant up or downregulated circRNAs identified in adenoma and tumor analyses. **B** Scatter plot of the correlation levels of differentially expressed circRNAs between log2FC computed in the analysis of adenoma and tumor samples. **C** Boxplots reporting the concentration (copies/μL) of selected circRNAs in lesion and adjacent tissue samples measured by ddPCR (*p < 0.05 by Paired Wilcoxon Rank-Sum test). **D** Heatmap of the hierarchical clustering analysis of Z-score-normalized circRNA levels in all the analyzed samples. **E** Dot plot of circRNA differential expression analysis between lesions (adenoma (left) and CRC (right)) and their corresponding adjacent mucosa in patients stratified for different clinical and molecular data. Dot size is proportional to p-value, while the color code reflects the log2FC expression levels

A paired differential expression analysis showed a widespread decrease of circRNA levels in tumor tissue compared with adjacent mucosa (3,268 circRNAs downregulated, 66%) (Fig. 2A and Table S1A). In most of the chromosomes, the number of downregulated circRNAs was higher than those upregulated (Figure S1C). In tumor samples, 34 of the identified circRNAs were significantly differentially expressed (median BSJ-aligned reads > 10 in at least one sample class, adj. *p* < 0.05), with 33 of them downregulated in tumor tissue compared to adjacent mucosa (Fig. 2A). The circRNAs with the most significant decrease were circEPB41L2(2,4), circPLCE1(2), and chr14:34551714|34554912 (Fig. 2A). Conversely, only one circRNA—circVRK1(2,11)—was upregulated in tumor samples with respect to adjacent mucosa (Fig. 2A).

In adenoma samples, two circRNAs (circRELL1(4,6) and circLINC00632(5)) significantly downregulated in tumor tissues when compared to the adjacent mucosa were confirmed (Fig. 2A and Table S1B). Despite not reaching a statistical significance, the fold changes of the other differentially expressed circRNAs observed in tumors were coherent with those of adenomas (ρ = 0.52, *p* < 0.01) (Fig. 2B).

Significantly lower levels of both circLINC00632(5) and circLPAR1(2,3) in tumor and adenoma tissues in comparison with matched mucosa were also validated by ddPCR (*p* < 0.05 by paired Wilcoxon Rank-Sum test) (Fig. 2C). Conversely, quantitative analysis for circVRK1(2,11) reflected the higher levels observed in tumors compared to adjacent mucosa, though this difference did not reach statistical significance (Fig. 2C). For all three tested circRNAs, the ddPCR levels and the number of normalized BJS reads were significantly correlated (*p* < 0.05) (Figure S1D).

CircRNA quantification by RNA-Seq on tumor tissues from the independent COLOBIOME cohort [25] of 159 CRC patients confirmed that all the differentially expressed circRNAs of the IT-cohort (Table S1C) were detectable. Among them, circSMARCA5(15,16) was detected in the highest number of samples.

Seventeen of the 34 dysregulated circRNAs in the IT-cohort were also detected in the Garcia-Rodriguez et al. [20] study. The analysis of the circRNA levels in overlap with our dataset confirmed a decreased expression in the tumor compared to adjacent normal tissue, including the decrease of circLINC00632(5) and circLPAR1(2,3). Similarly, in cancer cells, circRNA levels were reduced compared with stroma and senescent muscle cells (Table S1C). In another study, 14 of the 34 circRNAs were significantly altered in tumor compared to adjacent mucosa, and again, they were confirmed as mostly downregulated (*n* = 13), including circLINC00632(5) [17]. Overlapping the data from a third study on circRNA profiles in CRC tissue and adjacent/normal mucosa [15], 32 out 34 circRNAs of our study were also detectable in this dataset (circLINC00632(5), circHIPK3(2), and circCSNK1G(2,4) were the most expressed circRNAs), and 27 were also differentially expressed (*p* < 0.05) with a coherent log2FC (Table S1C). Among them, circLPAR1 (2,3), circLINC00632(5) and circPLCE1(2) levels were again lower in samples from tumors with respect to adjacent mucosa, while circVRK1(2,11) was upregulated in tumors. The prediction of single-cell circRNA localization from the same study reported that none of the differentially expressed circRNAs showed a specific cell-type expression, even though they were detected in both epithelial and stroma cells (Table S1C).

Clustering analysis highlighted three circRNAs (circEPB41L2(2,4), circWDR78(2,4), and circLPAR1(2,3)) whose levels were homogenously higher in most of the adjacent tissues analyzed with respect to tumor/adenoma (Fig. 2D). This result was confirmed by a patient-wise analysis showing circEPB41L2(2,4) and circLPAR1(2,3) as those circRNAs whose levels decreased in the highest number of tumor samples in comparison with adjacent tissue pairs (Figure S1E).

### A decrease of circRNA levels characterized early-stage tumors

Paired differential analysis between the tumor and matched adjacent mucosa stratified by different clinical and molecular features showed distinct patterns of dysregulation. Specifically, even though the differentially expressed circRNAs were equally downregulated (*p* < 0.05) in tumors stratified by location (colon or rectum), their decrease was prevalent in early-stage tumors (stage 0-I and II) with 28 circRNAs (82.4%) significantly downregulated (Fig. 2E). Accordingly, in AA patients, there was a prevalence of circRNA downregulation (*n* = 10) with circEPB41L2(2,4), circLINC00632(5), circLPAR1(2,3), and circRELL1(4,6) showing the strongest decrease. In nAA, circLINC00632(5) and circLPAR1(2,3) were confirmed as downregulated compared to adjacent tissue, while circRHOBTB(6,7) showed increased levels (Fig. 2E). However, given the low number of nAA subjects, these last results should be carefully considered.

Investigating circRNA profiles concerning other clinical and molecular features, a prevalence of circRNA downregulation in *APC*-mutated tumors and those without *KRAS* mutation or lacking a mucinous component was observed (Fig. 2E). The most downregulated circRNAs (particularly circLPAR1(2,3), circWDR78(2,4), circEPB41L2(2,4) and circLINC00632(5)) clustered together and were also associated with similar expression profiles with respect to patients’ clinical data (Fig. 2D-E). The expression analysis with respect to the estimated Consensus Molecular Subtypes (CMS) [42] showed 29 significantly downregulated circRNAs in tumors classified as CMS2 (canonical), including circLINC00632(5), circEPB41L2(2,4), and circWDR78(2,4). Significantly lower circRNA expression was also observed in CMS3 (metabolic) and CMS4 (mesenchymal) tumor subtypes, while no circRNA was significantly associated with high MSI and CMS1 (MSI immune) (Figure S1F).

When patients were stratified based on the median circRNA levels, it was observed that lower levels of circYY1AP1(4,5), circUBXN7(3,5), and circLPAR1(2,3) were significantly associated with better overall survival (age and sex-adjusted Cox-regression test *p* < 0.05; Figure S1G).

### CircRNA host genes are downregulated in tumor tissues

**Fig. 3:**
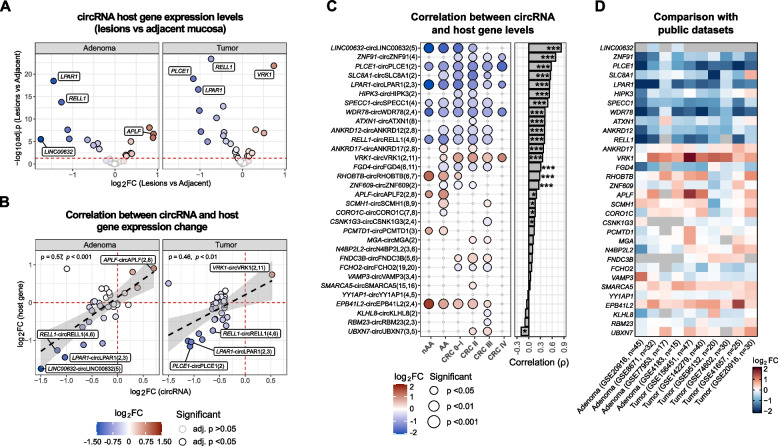
CircRNA host genes are downregulated in tumor tissues. **A** Volcano plots of the differential expression analysis of the differentially expressed circRNA host genes levels in lesions with respect to adjacent mucosa. Red dashed line indicates adj. *p* < 0.05. Black border identifies significant results. **B** Scatter plots of the relationships between log2FCs computed for the differentially expressed circRNAs and their host genes. The label highlights the most significant up/downregulated circRNA host genes. **C** Dot plot of the differential expression of circRNA host gene levels between lesions and adjacent mucosa samples in patients stratified according to disease stage. The dot size is proportional to the analysis significance. On the right, bar plot of the results of the Spearman correlation analysis between circRNA and their host gene levels. *p < 0.05; ***p < 0.001. **D** Heatmap reporting the differences in circRNA host gene levels (log2FC) between lesions and adjacent mucosa considering samples from ten public independent datasets

Since most of the dysregulated circRNAs were exonic (*n* = 32), their expression levels may reflect those of their corresponding host genes. Therefore, the expression levels of the linear isoforms were also investigated (Fig. 3A). Among the identified host genes, 23 (72%) were significantly dysregulated in tumor samples with respect to adjacent mucosa (16 downregulated and seven upregulated). The differential expression of 17 genes (16 downregulated and one upregulated) was consistent with the expression patterns of their associated circRNAs, including *LINC00632*, *LPAR1*, *RELL1*, *PLCE1,* and *VRK1* (Fig. 3A-B and Table S1B).

As confirmation of what observed, a correlation analysis showed a prevalence of positive associations between the levels of the differentially expressed circRNAs and those of their linear isoforms (Fig. 3C right panel and Table S1B), with 20 circular/linear isoform levels significantly related (adj. *p* < 0.05). The strongest circular/linear isoform correlation was observed for circLINC00632(5) (ρ = 0.83), followed by circZNF91(4) (ρ = 0.68), circPLCE1(2) (ρ = 0.57), circSLC8A1(2) (ρ = 0.55), and circLPAR1(2,3) (ρ = 0.53). Five inverse correlations were also observed, albeit only one was statistically significant (circUBXN7(3,5)-*UBXN7*, ρ = −0.19, adj. *p* < 0.001). The circular/linear isoform pairs characterized by the strongest correlations were also significantly downregulated in early/advanced tumor stages and precancerous lesions **(**Fig. 3C, left panel).

Nine public datasets reporting gene expression levels in tumor/adenoma tissues matched with adjacent mucosa were investigated to independently test the observed circular-linear isoform concomitant dysregulation (Fig. 3D). Expression changes were significantly coherent with those observed in our cohort (Fig. 3C left panel). For instance, *VRK1* showed a consistent upregulation in tumor/adenoma tissue with respect to adjacent mucosa, while *WDR78*, *LPAR1,* and *RELL1* (the top three downregulated circRNA host genes in our dataset) decreased.

To support these findings, a single-cell expression data analysis from Lee et al. [35] highlighted that, except for tumor cells classified as CMS4, circRNA host gene levels in cancer cells were significantly lower than those from normal epithelium or stromal cells (Figure S2A).

### RNA-binding proteins involved in circRNA biogenesis are downregulated in tumor tissues

**Fig. 4:**
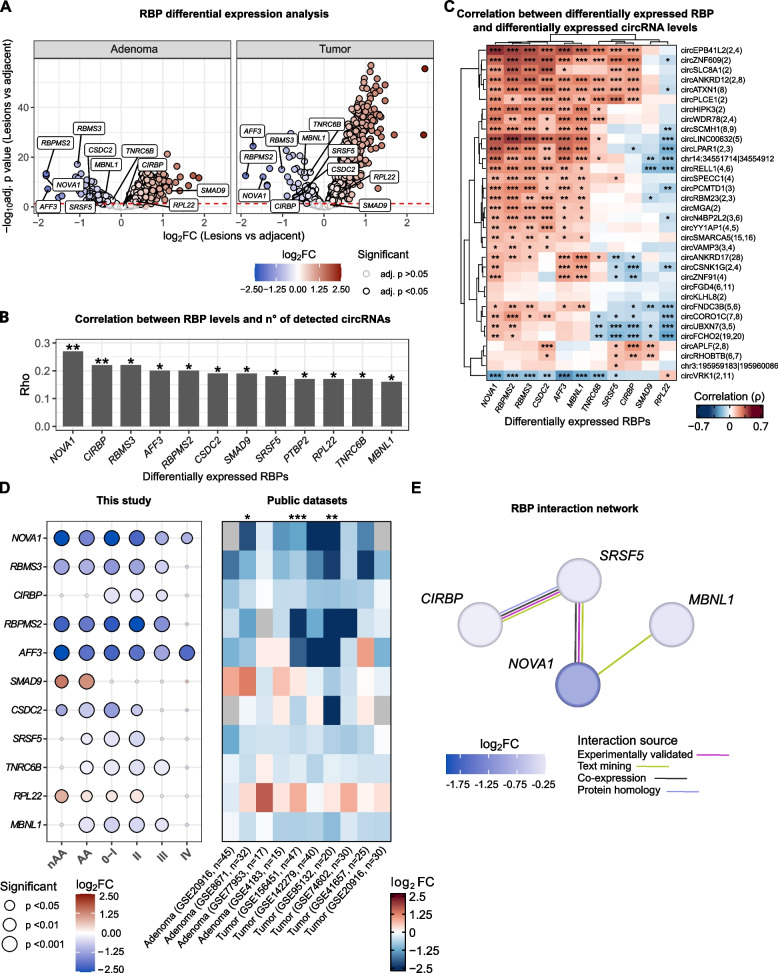
RNA-binding proteins involved in circRNA biogenesis are downregulated in tumor tissue. **A** Volcano plot of the differential expression analysis of RBP gene levels between lesions and adjacent mucosa. Black border highlights the significant results, while labels highlight the 11 RBPs whose levels significantly correlated with the number of detected circRNAs. **B** Bar plot of the correlation between differentially expressed RBP gene levels and the number of detected circRNAs in each sample. *p < 0.05; **p < 0.01. **C** Heatmap of the correlation between levels of RBP genes and those of the differentially expressed circRNAs. *p < 0.05; **p < 0.01; ***p < 0.001. **D** Dot plot of the differential expression of RBP genes between lesion and adjacent mucosa stratified according to disease stage. Size is proportional to the analysis significance. On the right, the heatmap reports the results of the differential expression analysis (log2FC) computed using 10 independent public datasets. *p < 0.05; **p < 0.01; ***p < 0.001. **E** Interaction network of circRNA-correlated RBPs. Node color reflects the log2FC from the differential expression analysis, while the link color identifies the type of supporting evidence

To investigate whether the observed circRNA dysregulation was related to impaired co- or post-transcriptional regulatory events regulated by specific RBPs, an evaluation of RBP-encoding mRNA profiles in tumor and adenoma samples was performed. In our dataset, paired differential expression analysis showed 1,177 RBPs significantly dysregulated (adj. *p* < 0.05 and median TPM > 1) in adenoma/tumor samples. Of those, 1,035 were upregulated and 142 downregulated (Fig. 4A and Table S2A). Correlation analysis was performed between each RBP-encoding gene level and the number of detected circRNAs in each sample (Fig. 4B and Table S2A). Eleven dysregulated RBPs associated with a significant positive correlation (adj. *p* < 0.05) were identified. Among them, *NOVA1* was the RBP-coding gene with the strongest correlation (ρ = 0.27), followed by *RBMS3* (ρ = 0.22), *CIRBP* (ρ = 0.22), and *RBPMS2* (ρ = 0.20) (Fig. 4B and Table S2A). The correlation between the levels of these RBPs and those of the 34 differentially expressed circRNAs showed a prevalence of positive associations (adj. *p* < 0.05) involving mostly *NOVA1*, *RBPMS2*, *RBMS3*, and *CSDC2* (Fig. 4C). Motif analysis showed the presence of *NOVA1*, *RBMS3*, and *MBNL1* motifs close to back-spliced junctions (Table S2B). In particular, the *RBMS3* motif was identified for 18 dysregulated circRNAs (14 upstream of the BSJ, four downstream), followed by *NOVA1* (*n* = 15) and *MBNL1* (*n* = 13) (Figure S2B).

Stratification analysis based on patient clinical data revealed that the levels of these RBP-coding genes were significantly lower in colorectal lesions compared to adjacent mucosa samples in most of the disease stages (Fig. 4D, left panel). *NOVA1* and *AFF3* were significantly downregulated in all the disease stages and precancer lesions. Conversely, *SMAD9* showed a significant upregulation in adenoma, while *CIRBP* was downregulated only in tumors. The dysregulation trend was coherently observed in the nine independent public datasets already included in the study (Fig. 4D, right panel).

A protein–protein network analysis among candidate RBPs was performed to evaluate candidate complexes involved in the biogenesis of the differentially expressed circRNAs. The analysis showed Serine and Arginine Rich Splicing Factor 5 (SRSF5) as the most connected RPB, interacting with other RBPs coded by *CIRBP*, *NOVA1* and *MBNL1* genes (Fig. 4E).

Despite analysis of retrieved single-cell data did not show a significant difference in their expression between stroma and tumor cells, RBP levels in tumor cells were generally lower with respect to those of epithelial and stromal cells from normal colonic tissue (Figure S2A).

### The levels of circRNAs inversely correlate with proliferation-related pathways

**Fig. 5:**
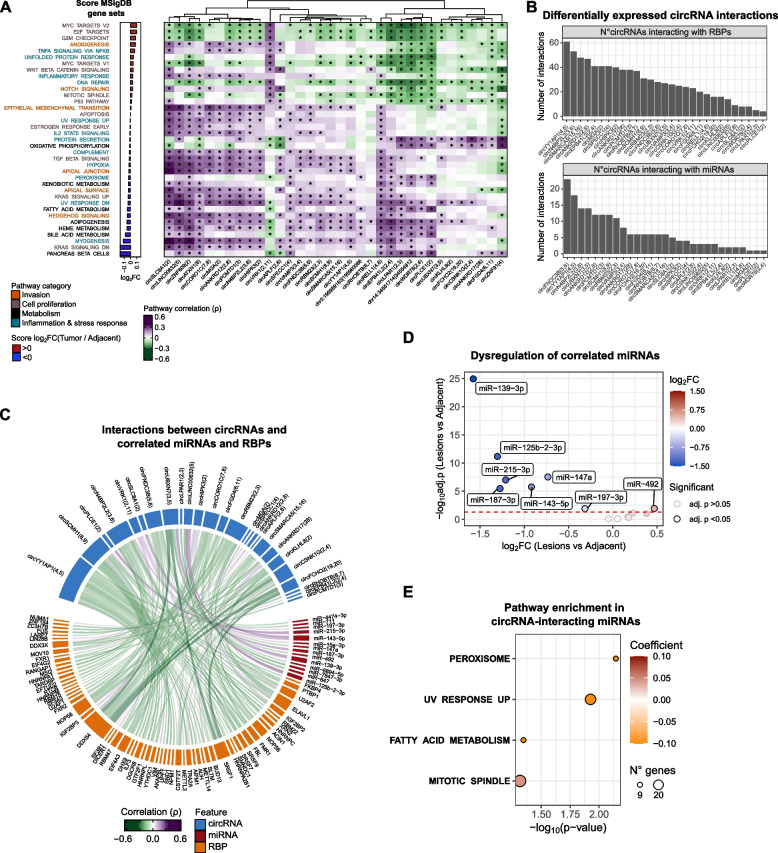
Dysregulated circRNA levels inversely correlate with proliferation-related pathways. **A** Bar plot of the log2FC of MSigDB hallmark gene set scores computed between lesions and adjacent mucosa samples (left) and heatmap of the correlation between differentially expressed circRNA levels and hallmark pathway scores (right; *p < 0.05). The heatmap color gradient reflects the correlation coefficient (green for negatively correlated and purple for positively correlated). **B** Bar plot reporting the number of interactions with RBPs (up) and miRNAs (down) predicted for each dysregulated circRNA. **C** Circos plot of the significant and coherent correlations between differentially expressed circRNAs and interacting miRNAs and RBP genes (blue, red, and orange arches, respectively). Edge color and intensity reflect the correlation coefficient. **D** Volcano plot of the differential levels of miRNAs interacting with circRNAs when analyzed in lesions compared to adjacent mucosa. Labeled dots identify the differentially expressed miRNAs. The red dashed line indicates adj. *p* < 0.05. **E** Dot plot of hallmark gene set enrichment analysis considering the validated targets of dysregulated miRNAs interacting with circRNAs. The dot size is proportional to the number of target genes, while the color reflects the predicted pathway activation levels (Coefficient)

A functional analysis was performed to identify pathways which may be affected by circRNA dysregulation. Since circRNAs are not ontologically annotated as protein-coding genes, a guilt-by-association analysis was performed between circRNA levels and a single-sample enrichment score of specific molecular pathways (see Material and Methods for more details). Specifically, the single-sample score of 1,701 MSigDB hallmark gene sets [36] was computed for each tumor and adjacent mucosa sample using the RNA-Seq data. As expected, based on the resulting scores, pathways related to cell proliferation (e.g., *E2F TARGET*, *G2M CHECKPOINT*, *MITOTIC SPINDLE*, *MYC TARGETS*) were enriched in tumors, while metabolism-related terms (e.g., *FATTY ACID METABOLISM*, *BILE ACID METABOLISM*, *ADIPOGENESIS*) were enriched in adjacent mucosa samples (Fig. 5A and Table S3A).

Correlation analysis between the above scores and differentially expressed circRNA levels showed that the latter negatively correlated with pathways scoring higher in lesions than in adjacent mucosa (pathway score log2FC > 0). Conversely, pathways scoring lower in the lesion samples were positively correlated with the circRNA levels (Fig. 5A and Table S4A). In this respect, strongly downregulated circRNAs (i.e., circLINC00632(5), circRELL1(4,6), circPLCE1(2) (ρ = 0.57), circLPAR1(2,3), and circEPB41L2(2,4)) were correlated with metabolism-related pathways while inversely related with proliferation-related terms (Fig. 5A). In contrast, circVRK1(2,11) inversely correlated with metabolism-related pathways and positively correlated with proliferation-related pathways (Fig. 5A).

### A network of miRNAs and RBPs is predicted to interact with the differentially expressed circRNAs

To inspect regulatory events involving the observed dysregulated circRNAs, the annotations of their interactions with miRNAs and RBPs were retrieved from *CircNet* [38]. Twenty-three circRNAs were predicted to interact with 166 and 117 miRNAs and RBPs, respectively (Fig. 5B and Table S3B). The circRNAs characterized by the highest number of interactions were circYY1AP1(*n* = 61), circN4BP2L2(3,6) (*n* = 53), and circSMARCA5(15,16) (*n* = 48). miR-143-5p, miR-215-3p, and miR-298 (*n* = 3) were the most interacting miRNAs, while the most interacting RBPs were Insulin Like Growth Factor 2 mRNA Binding Protein 2 (IGF2BP2, *n* = 29), Eukaryotic Translation Initiation Factor 4A3 (EIF4A3, *n* = 27), and Argonaute 2 (AGO2, *n* = 25) (Figure S2C). To characterize these interactions, miRNA and RBP expression levels measured in the same tissue samples were analyzed and correlated. Focusing only on significant correlations (adj. *p* < 0.05) supported by coherent trends (see Materials and Methods section for details), 157 miRNA/RBP interactions with circRNAs were confirmed (Table S3C). Generally, RBP levels, mostly increased in tumor samples, were inversely related to those of the circRNAs (Fig. 5C and Table S3C). miRNA levels were either positively correlated with circRNA levels (nine significant correlations) or negatively (seven significant anti-correlations) (Fig. 5C and Table S3C). Globally, miRNAs were characterized by fewer associations than RBPs (Fig. 5C).

### Targets of miRNAs interacting with dysregulated circRNAs are involved in inflammatory and cell-cycle pathways

Among the identified circRNA-interacting miRNAs, eight were significantly differentially expressed (one up- and seven downregulated) between tumor and adjacent mucosa (Fig. 5D and Table S3D) with miR-139-3p (adj. *p* = 1.14e-25, log2FC = −1.58), miR-125b-2-3p (adj. *p* = 6.73e-12, log2FC = −1.30) and miR-147a (adj. *p* = 2.97e-08, log2FC = −0.73) being the most significantly dysregulated.

MSigDB hallmark gene set analysis considering the validated targets of these differentially expressed miRNAs showed an enrichment of terms related to metabolism, cell duplication, and stress/inflammation response (Fig. 5E and Tables S3E-F). All genes involved in these pathways represented validated targets of correlated miRNAs, particularly miR-143-5p and miR-139-3p (Table S3F). These miRNAs were associated with negative correlations with the differentially expressed circRNA levels (40%), particularly with circSLC8A1(2) and circFNDC3B(5,6) (Table S3F).

## Discussion

The present study dissected the expression profiles of circRNAs in paired tumor/precancer and adjacent mucosa tissues observing a genome-wide decrease of these RNA molecules in CRC. An integrative analysis revealed that the decreased expression of RBPs involved in back-splicing and downregulation of host gene transcription could partially explain the reduced circRNA levels detected in the tumor tissues. Interestingly, tumor molecular classification showed that circRNAs were prevalently downregulated in the early disease stages, particularly stage 0-I tumors with *APC* but no *KRAS* mutations and MSI-stable CMS2 tumors, the molecular subtype characterized by *WNT* and *MYC* mutation and an epithelial-like phenotype [42].

Previous studies, in line with our findings of a widespread decrease in circRNA levels, have shown that circRNA expression is generally lower in primary and metastatic tumors compared to normal or senescent cells [17, 20]. A lower number of circRNAs in tumor samples was also recently reported by Li and colleagues by comparing the circRNA profiles among tumor, adjacent, and normal colonic mucosa samples [15]. Interestingly, despite the author observed circRNA upregulated in tumor samples, the in vitro silencing of two circRNAs, also downregulated in our data (circPLCE1(2) and circLPAR1(2,3)), increased the proliferation of LoVo CRC cell lines. These results suggest a direct regulatory activity of identified circRNAs in the regulation of cancer cell proliferation.

Our study further explored the molecular events involved in the downregulation of circRNAs, such as the repression of transcription of certain circRNA host genes and RBPs that promote back-splicing events. As expected, a positive correlation between circular and linear isoform levels was prevalently observed, consistent with the circRNA synthesis as a circularization event of the linear transcript. Among 32 circRNA host genes identified within the 34 differentially expressed circRNAs, 11 were significantly downregulated in tumor tissue, including six (*LINC00632*, *PLCE1*, *LPAR1*, *SPECC1*, *WDR78*, and *RELL1*) that also showed decreased expression in adenoma samples. This latter finding suggests that such downregulation may be an early event in colorectal tumorigenesis. Interestingly, *PLCE1*, *LPAR1,* and *LINC00632* have previously been reported as downregulated genes in CRC [43–46]. For instance, Wang and colleagues observed a reduction in *PLCE1* expression in tumor tissues, hypothesizing that it could be linked to tumor aggressiveness [43]. Moreover, Martins and colleagues demonstrated the tumor suppressor activity of *PLCE1* in *KRAS*-mutated tumor models [44] while analysis of single-cell data from Lee et al. [35] reinforced the detectability of *PLCE1* in both epithelial and stromal cells, supporting a tumor-specific downregulation. Interestingly, another study reported lower levels of circPLCE1(2) in tumor tissues, but higher levels of this circRNA were associated with a better prognosis [47].

Song and colleagues showed that the *LPAR1* gene was highly expressed in normal colorectal tissue and downregulated in tumor cells after the analyses of more than 2,000 transcriptomes of CRC samples [45]. Moreover, the associated circRNA, circLPAR1(2,3), was downregulated in tumor tissues matched with adjacent mucosa samples from 112 CRC patients, and lower levels of this circRNA were associated with worse patient overall survival [48]. The decrease of *LPAR1* levels was also reported in public single-cell data from Lee and colleagues [35], showing a prevalent expression in tumor stroma.

Zhu and colleagues reported in CRC a downregulation of *LINC00632* [46] whose encoded circRNA circLINC00632(5), also known as ciRS-7 or CDR1as, has been previously deeply characterized for its impressive number of miRNA-binding sites [49]. However, in CRC patient samples, a stromal-specific localization of both *LINC00632* and the encoded circRNA was reported, suggesting that the observed downregulation in tumor tissues is an indirect effect of the lower percentage of stromal cells [50]. More recently, in mouse models, the expression of circLINC00632 has been reported in the intestinal epithelium, while its activity in inhibiting the proliferation of intestinal cells during an injury-induced epithelial regeneration has been demonstrated [51]. A similar activity in regulating intestinal epithelium homeostasis was reported for circHIPK3(2) [52], another circRNA downregulated in tumor tissue from our dataset. Altogether, these data suggest that for specific circRNAs a role in the early phase of carcinogenesis might be linked with the perturbation of their physiological activity involving stromal and epithelial cells.

The observed deregulation of RBPs involved in circRNA biogenesis further supports regulatory events linked with decreased circRNA levels. Previous studies showed that > 1,000 RBPs are dysregulated in tumor samples, including several known to influence circRNA production and availability [19]. In the present study, the expression levels of 11 differentially expressed RBP-coding genes were significantly correlated with the number of detected circRNAs. This subset was significantly downregulated in both tumor and precancerous lesions, suggesting that their decreased expression may impair circRNA production. Among these RBPs, *NOVA1*, *RBMS3*, and *MBNL1* emerged as particularly compelling candidates due to the presence of their binding motif near back-splicing sites.

*NOVA1* encodes for the NOVA Alternative Splicing Regulator 1 (Nova1) whose activity was extensively characterized in neuronal tissue and found to be dysregulated in various tumors [53]. Nova1 has been identified as a regulator of circRNA biosynthesis [54]; however, in mouse models, the knock-out of Nova2, but not of Nova1, impairs the neuronal circRNA biogenesis, suggesting a tissue-specific function of these proteins [55]. The altered expression of *NOVA1* promotes tumor progression in CRC patients by modulating JAK2/STAT3 pathway [56]. The role of *RBMS3* in circRNA biogenesis has also been established [57, 58], and its downregulation has been previously reported in CRC [59]. Muscleblind Like Splicing Regulator 1 (MBNL1) is one of the first validated RBP involved in circRNA biogenesis, mainly in circularizing its own mRNA [60]. Indeed, MBNL1 associates the second exon of *MBNL1* mRNA, promoting its back-splicing in competition with the linear splicing events. In addition, Navvabi and colleagues found downregulation of this gene in tumors compared to normal mucosa in a cohort of 108 CRC patients [61]. Noteworthy, *MBNL1* was highly expressed in both tumor and stroma cells, while *NOVA1* and *RBMS3* were dominantly expressed in tumor stroma (Table S1D).

Limited evidence supports the roles of three RBPs identified in our study, SRSF5, RPL22, and TNRC6B, in the regulation of circRNA. A large-scale analysis of CLIP data by Khao and colleagues highlighted the involvement of SRSF5 in circRNA biogenesis and their findings indicated that silencing SRSF5 significantly impaired circRNA biosynthesis [19]. Similar to MBNL1, RPL22 plays a role in the circularization of its own primary transcript. Notably, RPL22 associates to a stem-loop structure located on exon two of the linear *RPL22* transcript, thus promoting its circularization and facilitating the production of the circRPL22 [62]. Conversely, the role of TNRC6B in circRNA biogenesis remains controversial. Jia and colleagues suggested that TNRC6B may be involved in circRNA degradation, with their results showing that depletion of TNRC6B led to the accumulation of various circRNAs [63]. Based on our literature search, there is currently no significant data available regarding the other RBPs identified in our study. Further experiments are needed to clarify their roles in circRNA biogenesis.

The functional guilt-by-association analysis of the differentially expressed circRNAs showed significant correlations with cell proliferation pathways, including those of c-Myc and β-Catenin, both commonly altered in CRC [64]. Analysis of RBPs interacting with altered circRNAs showed a widespread inverse correlation between their levels. IGF2BP2, known for its role in circRNA export [65], and EIF4A3, a key component of the exon-junction complex involved in mRNA translation [66], were among the RBPs with the highest number of interactions. In this respect, circRNA translation into small peptides has previously been reported [10, 11] and, interestingly, our study identified Open-Reading Frame (ORF) in most of the dysregulated circRNAs (Figure S2D and Table S3G). Recent findings showed that cryptic peptides encoded from circRNAs can stimulate a CD4 + and CD8 + T cell-mediated anti-tumor immunity [10]. This raises intriguing questions about whether the early circRNA decrease in tumor cells could contribute to tumor immune evasion, an area worth exploring in future studies.

The correlation analysis between differentially expressed circRNAs and their interacting miRNAs revealed both positive and negative associations. The most correlated miRNAs were also downregulated in our data and included miR-143-5p, a miRNA overexpressed in the intestinal tissue, particularly in the mesenchymal cells [67]. The downregulation of miR-143 in CRC is controversial and seems mainly linked to a stromal localization [68]. While further experiments are needed, the prevalent downregulation observed in our data suggests that some circRNA-miRNA regulatory axes might represent a growth disadvantage of proliferation cells, particularly in the early phase of carcinogenesis. This aligns with evidence that 3’UTR shortening is a hallmark of the cancer cell transcriptome [69, 70] and that certain oncogenic mutations can impair the AGO2 activity [71], impacting miRNA regulation.

The observed decrease in circRNA levels may be partially explained by the rapid cell division typical of tumor cells that could lead to a dilution of the cytoplasmic content, including circRNAs [20, 22]. Garcia-Rodriguez and colleagues found similar reduction in circRNA levels when comparing cancer cells to normal adjacent tissue, particularly in senescent muscle cells. This further supports the global circRNA dilution and reduction hypothesis during cell proliferation [20]. Interestingly, out of 48 dysregulated circRNAs that Garcia-Rodriguez and colleagues found, 16 overlapped with those identified in our study, including circLINC00632(5) and circEPB41L2(2,4) and single-cell analysis showed that these circRNAs are not confined to stromal compartment [17]. In addition, the analysis of single-cell data from Lee et al., [35] showed that 25 circRNA host genes out of the 32 identified in our study were expressed in normal and, with lower levels, in tumor epithelial cells, while only seven (*LINC00632*, *SLC8A1*, *LPAR1*, *WDR78*, *RELL1*, *APLF*, *RHOBTB1*) had a prevalent expression in the stromal cells (Table S1D). Further studies are needed to determine if circRNA decrease is a direct consequence of cellular transformation or an indirect effect of tumor cell proliferation. These data, in relationship with the expression of the host gene regulating RBPs, support the idea that multiple synergic factors may converge in decreasing circRNA levels in cancer cells.

We are aware that our study has limitations that require further investigations. For instance, some of the patient classes (nAA, stage 0, and IV CRC) were poorly represented limiting possible conclusions on very early-stage diseases or metastatic tumors. In addition, circRNA profiling was generated from data of total RNA-Seq without RNase digestion, allowing the detection of highly expressed molecules but losing the resolution of lowly expressed circRNAs. However, the employment of CIRI2 to profile circRNAs from RNA-Seq data is one of the most sensitive methods for the identification of circRNAs [72]. Despite this, to our knowledge, the present study represents one of the few studies that deeply characterized circular RNA transcription on a large group of CRC or precancerous patients with available lesions and matched adjacent mucosa data. Indeed, most of the published circRNA studies refer to only one molecule or, more frequently, profile a very limited set of patients whose size is too small to provide a reliable overview of circRNA expression in this cancer.

## Conclusions

We presented an integrative analysis of circRNA profiles in tissue samples from a large cohort of CRC and adenoma patients. Our results demonstrate that circRNA levels decreased in samples from lesions with respect to adjacent mucosa already in precancerous lesions and early-stage CRC. We also observed a coherent dysregulation of circRNA host genes and RBPs involved in back-splicing and, in addition to cytoplasmic dilution, that may be a cause for the decreased circRNA levels that were observed in CRC. In conclusion, this work may help understand the biological role of circRNAs and their dysregulation in CRC, highlighting their possible clinical relevance in this oncologic disease.

## Supplementary Information

Supplementary Material 1: Document S1. Figures S1–S2.

Supplementary Material 2: Table S1. Excel file containing additional data too large to fit in a PDF, related to Figure 2 and 3.

Supplementary Material 3: Table S2. Excel file containing even more data too large to fit in a PDF, related to Figure 4.

Supplementary Material 4: Table S3. Excel file containing even more data too large to fit in a PDF, related to Figure 5.

## Data Availability

The datasets used and/or analyzed during the current study are available from the corresponding author on reasonable request.

## Abbreviations

circRNA: Circular RNA
CRC: Colorectal Cancer
RNA-Seq: RNA Sequencing
nAA: Non-Advanced Adenoma
AA: Advanced Adenoma
miRNA: Micro RNA
RBP: RNA-binding Protein
ddPCR: Droplets digital Polymerase Chain Reaction
CMS: Consensus Molecular Subtype
mcCRC: CRC with mucinous component
mCRC: Mucinous CRC
SD: Standard Deviation
TPM: Transcripts Per Million
adj.p: Adjusted pvalue
rRNA: Ribosomal RNA
ORF: Open Reading Frame
MBNL1: Muscleblind Like Splicing Regulator 1
NOVA1: NOVA Alternative Splicing Regulator 1
EIF4A3: Eukaryotic Translation Initiation Factor 4A3
AGO2: Argonaute 2
IGF2BP2: Insulin Like Growth Factor 2 mRNA Binding Protein 2
SRSF5: Serine and Arginine Rich Splicing Factor 5
