# Supplementary material for: Multiple regulatory events contribute to a widespread circular RNA downregulation in precancer and early stage of colorectal cancer development
Source: Biomark Res. 2025 Feb 20;13:30. doi: 10.1186/s40364-025-00744-8 (PMC11844049; doi:10.1186/s40364-025-00744-8)
Supplement: Supplementary file 1 — Supplementary Material 1: Document S1. Figures S1–S2. [file 40364_2025_744_MOESM1_ESM.docx]

**
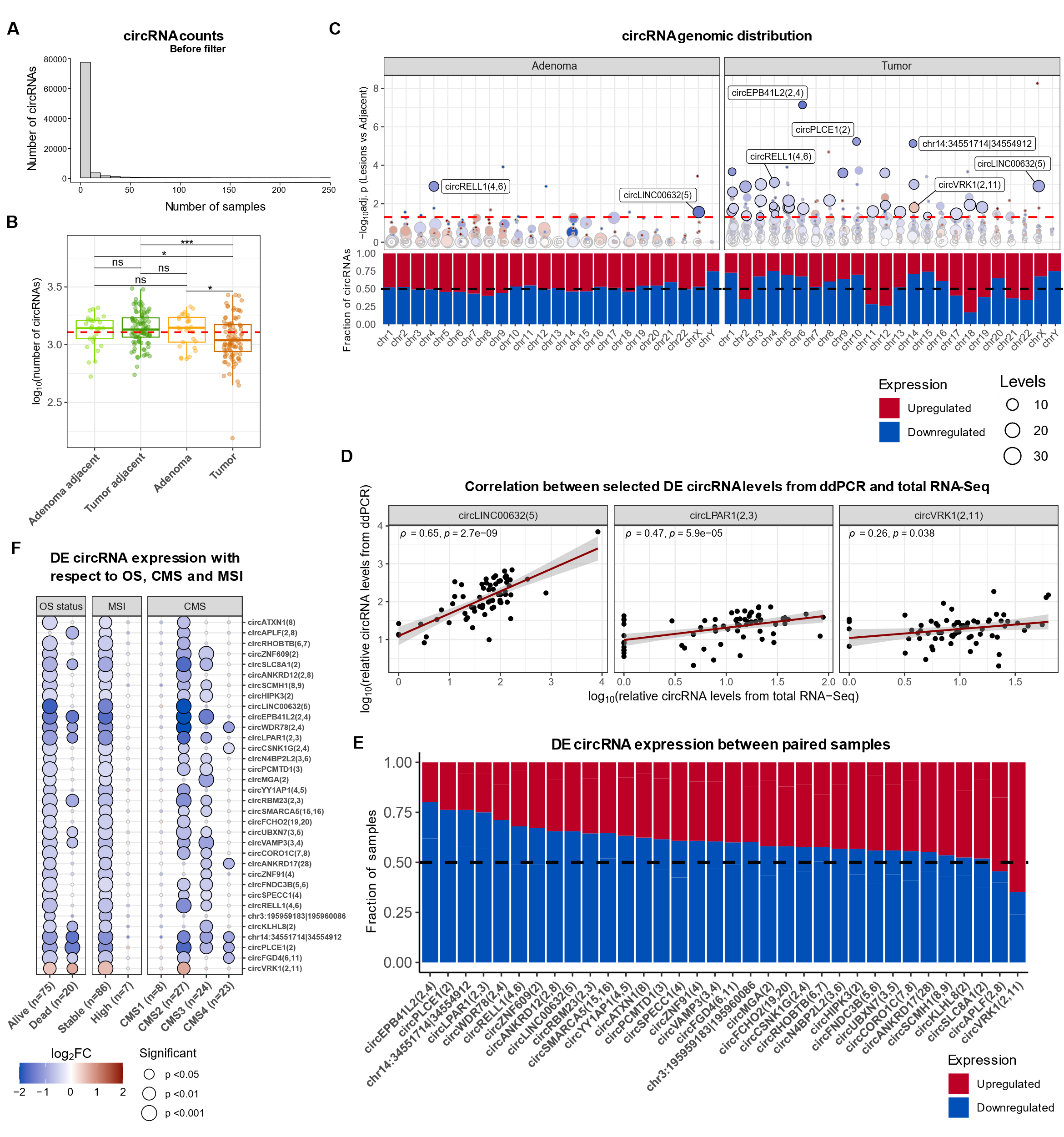
Supplementary Figures**

**Figure S1. A)** Distribution of circRNA count before the application of *NearZeroVar* function to exclude low expressed molecules. **B)** Box plots of the number of circRNAs in lesions and adjacent mucosa samples (*p<0.05; ***p<0.001). **C)** Manhattan plots of the genomic distribution of the detected circRNAs. The dot color reflects the log2FC, while the size is proportional to the median levels. The label highlights the two overlapping dysregulated circRNAs in adenoma and tumor, as well as the most significant up and downregulated differentially expressed circRNAs observed in tumor samples. **D)** Scatterplot of three circRNA levels measured by ddPCR (y-axis) or total RNA-Seq (x-axis). Spearman correlation analysis results are reported. **E)** Bar plot showing the fraction of samples with upregulated (red) and downregulated (blue) circRNAs between lesions and adjacent tissue. **F)** Dot plot showing the dysregulated circRNA levels in samples stratified for overall survival, CMS and MSI. The dot size is proportional to the significance of the analysis, while the color code reflects log2FC.

**
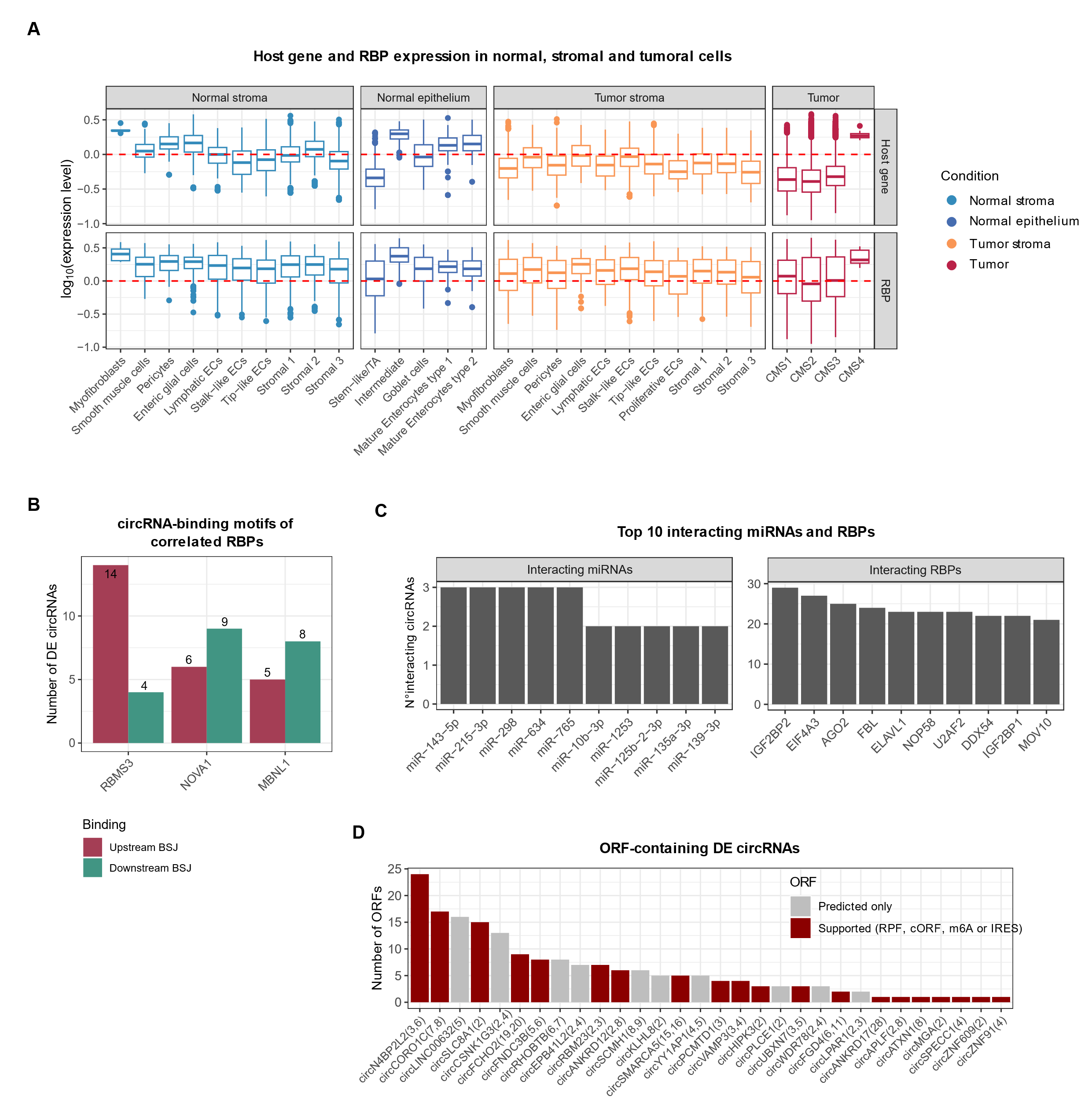
**

**Figure S2. A)** Box plots reporting the levels of circRNA host genes (top) and RBPs involved in circRNA biogenesis (bottom) in single cell populations from healthy (epithelium and stroma) and tumor tissue (stroma and CRC). Box color reflects the different sample condition, while horizontal red dashed line highlights the median expression. **B)** Bar plot reporting the number of circRNAs associated with RBPs containing a circRNA-binding motif. Red color reflects the motifs upstream of the BSJ, while teal reflects those downstream of the BSJ. **C)** Bar plots reporting the top ten miRNAs (left) and RBPs (right) interacting with circRNAs. Features are ordered according to the number of predicted interactions. **D)** Bar plot showing the counts of predicted ORFs within the differentially expressed circRNAs identified in this study. The red color highlights the supported ORFs, the grey color corresponds to the predicted ORF.

**Supplementary Tables**

Supplementary Tables are available in FigShare at the following links.

**Supplementary Table 1**

<https://doi.org/10.6084/m9.figshare.28270295.v1>

**Supplementary Table 2**

<https://doi.org/10.6084/m9.figshare.28270298.v1>

**Supplementary Table 3**

<https://doi.org/10.6084/m9.figshare.28270301.v1>
